# Geographic Analysis of Blood Lead Levels in New York State Children Born 1994–1997

**DOI:** 10.1289/ehp.7053

**Published:** 2004-08-18

**Authors:** Valerie B. Haley, Thomas O. Talbot

**Affiliations:** Bureau of Environmental and Occupational Epidemiology, New York State Department of Health, Troy, New York, USA

**Keywords:** geographic information systems, housing, lead poisoning, New York State, simultaneous autoregression, socioeconomic status, spatial autocorrelation

## Abstract

We examined the geographic distribution of the blood lead levels (BLLs) of 677,112 children born between 1994 and 1997 in New York State and screened before 2 years of age. Five percent of the children screened had BLLs higher than the current Centers for Disease Control and Prevention action level of 10 μg/dL. Rates were higher in upstate cities than in the New York City area. We modeled the relationship between BLLs and housing and socioeconomic characteristics at the ZIP code level. Older housing stock, a lower proportion of high school graduates, and a higher percentage of births to African-American mothers were the community characteristics most associated with elevated BLLs. Although the prevalence of children with elevated BLLs declined 44% between those born in 1994 and those born in 1997, the rate of improvement may be slowing down. Lead remains an environmental health problem in inner-city neighborhoods, particularly in upstate New York. We identified areas having a high prevalence of children with elevated BLLs. These communities can be targeted for educational and remediation programs. The model locates areas with a higher or lower prevalence of elevated BLLs than expected. These communities can be studied further at the individual level to better characterize the factors that contribute to these differences.

Although the rate of childhood lead poisoning has been decreasing because of the reduction in exposure to environmental lead sources and increased education, lead poisoning is still considered to be one of the most prevalent and preventable childhood health problems in New York State. New York State has the largest proportion (43%) and largest number (3.3 million) of housing units built before 1950 of any state (U.S. Census Bureau 2002). Because lead paint was not banned nationally until 1978 (U.S. Consumer Product Safety Commission 1977), older houses may still contain layers of lead paint that contaminate indoor dust when the paint deteriorates or spreads during renovation. Lead is also present in soil as a result of historical deposition from automobile exhaust, deteriorated paint, lead arsenate pesticide, and industrial and incinerator emissions. Other sources of lead exposure include plumbing, ceramicware, traditional remedies, and lead brought into the home from occupational and recreational exposures [Centers for Disease Control and Prevention (CDC) 1997].

Exposure to lead is significant because it damages the central nervous system and impairs learning and behavior even at low levels (Canfield et al. 2003). Young children are more vulnerable than adults for several reasons. They may ingest contaminated dust and soil as a result of normal mouthing activity. They also take in more lead as a proportion of body mass and absorb more lead than do adults (Mushak 1992). The toxicity of lead depends on the dose, the duration of exposure, and the developmental and nutritional susceptibility of the child (American Academy of Pediatrics 1998). The cost of the health effects of lead exposure is estimated to be $43.4 billion each year in the United States, much more than costs of other childhood diseases of environmental origin (Landrigan et al. 2002).

Locating areas with a high prevalence of children with elevated (blood lead levels) BLLs is important for identifying communities that could be targeted with educational and remediation programs. In this study, we examined the geographic distribution of elevated BLLs in New York State children born between 1994 and 1997. We also considered time trends. We used spatial regression to identify community characteristics associated with areas that exhibit a high prevalence of elevated lead values. The regression model identified areas that have higher rates of elevated BLLs than expected, after taking into account significant community characteristics. These areas can be examined more carefully to better characterize the local factors that contribute to lead exposure.

Previous studies have shown that elevated BLLs have been associated with housing and sociodemographic characteristics, including older housing stock, lower housing value, a higher proportion of children living below the poverty level, a lower proportion of high school graduates, a lower proportion of owner-occupied housing, a higher proportion of vacant housing, a higher proportion of households headed by a female, a higher proportion of minority births, a higher population density, and industrialization (Bailey et al. 1994; Griffith et al. 1998; Lanphear et al. 1998; Litaker et al. 2000; Miranda et al. 2002; Sargent et al. 1995, 1997; Talbot et al. 1998). In addition, studies have shown that children’s lead levels tend to be higher in the summer months (e.g., Yiin et al. 2000). These studies have several limitations that our analysis seeks to overcome.

Some of the earlier studies used only surrogate markers of BLLs (Bailey et al. 1994; Sargent et al. 1995). Other studies used data for only a small geographic area or time period (Griffith et al. 1998; Lanphear et al. 1998; Litaker et al. 2000; Miranda et al. 2002; Sargent et al. 1997), making it difficult to understand patterns over time in wider geographic areas. The present study is the largest population-based study in the literature to date. It includes New York City, the largest urban area in the country, with a wide spectrum of demographics, and considers a large proportion of children screened. The previously published study of New York State children (Talbot et al. 1998) used data for children born over a 2-year period, whereas the present study used data for children born over a 4-year period. This enabled us to perform the analysis at a finer geographic scale in some areas and to detect trends over time. We also sought to control for spatial autocorrelation that is inherent in childhood lead data (Griffith et al. 1998), to better estimate the variance in the data. Finally, our study only focused on children < 2 years of age. Other investigators studied children < 4–6 years of age. Younger children have different behavioral risk factors than do older children and tend to have higher BLLs (Brody et al. 1994; Griffith et al. 1998).

## Materials and Methods

The New York State Department of Health (NYSDOH) Laboratory Reporting System provided data on the BLLs of children born between 1994 and 1997 and screened before 2 years of age. New York State requires all health care providers to screen the blood of all 1- and 2-year-olds for lead (NYSDOH 2001). Data include the name, address, and date of birth of the child, the name and telephone number of a parent or guardian, the date of the test, the blood lead value, and the method by which the blood sample was obtained (finger-stick or venipuncture). More than 80 New York State certified laboratories reported results, but approximately 75% of the reports came from just 10 labs. For tests that were reported at below the detection limit, the average detection limit was 3 μg/dL. For the 34% of children who were screened more than once, we used the highest BLL done by venipuncture because these samples are less susceptible to environmental contamination than are fingerstick samples (Parsons et al. 1997). We used the highest fingerstick measure if no venipuncture measures were available for the child. In the final data set, 65% of the test results were from venipuncture, 31% were from fingerstick, and 4% had no method recorded.

The NYSDOH Bureau of Vital Statistics provided the number of births by race and ZIP code in New York State from 1994 to 1997. We estimated the screening rates as the number of children tested divided by the number of births.

We chose U.S. Postal Service ZIP codes, which are used for mail delivery, as the geographic level of analysis because they are readily available with the childhood lead data. There are 1,598 ZIP codes in New York State, with populations ranging from 0 to > 100,000. In cases where the child had a missing or invalid ZIP code, we used address-matching software to assign a ZIP code. To obtain valid ZIP codes in cases where no street or town information was available for the child, we matched the parent or guardian’s phone number to digital phone directories. We could not determine valid ZIP codes for 3% of the children screened for lead and for 0.2% of the birth records obtained from vital statistics files. These records were excluded from the analysis.

On the basis of a review of previous published work (Bailey et al. 1994; Griffith et al. 1998; Lanphear et al. 1998; Litaker et al. 2000; Miranda et al. 2002; Sargent et al. 1995, 1997; Talbot et al. 1998; Yiin et al. 2000), we selected the following variables for further study: the percentage of homes built before 1940 and 1950, the percentage of adults ≥25 years of age who did not receive a high school diploma, the percentage of children living below the poverty level, the percentage of vacant housing units, the percentage of the population that rents a home, the percentage of children screened in summer (July–September), population density, and the percentage of African-American births. We used the percentage of children < 5 years of age living below the poverty level rather than median household income because median household income does not focus specifically on households with young children, and once a household reaches a certain threshold income level, further increases in income may not lead to improved living conditions and reduced exposures. The percentage of results obtained by venipuncture was not included as a variable because it is related to the outcome measure; venipuncture is the recommended confirmatory test for an initial high BLL. Several sources provided the socioeconomic data because not all of the Census 2000 data were available at the time of analysis. Age of housing data for 1999 were obtained from Claritas, Inc. (Ithaca, NY). We obtained the socioeconomic data at the census block and block group level (U.S. Census Bureau 1991, 1992, 2001) because 1990 Census ZIP code population estimates and 1990 Census ZIP code boundaries did not conform to the more current and accurate ZIP code boundaries used in this study. We used a geographic information system (MapInfo Professional version 7.5; MapInfo, Inc., Troy, NY) to apportion the socioeconomic data based on population and the location of the block centroids. We estimated the proportion of African-American children in each ZIP code using the mother’s race from the birth certificate because it contained more complete race information than did the lead database.

Mapping data in small geographic areas such as ZIP codes often produces rates that vary widely when the population is small or the health outcome under analysis is rare. To obtain more stable numbers for the spatial analysis, we combined data for children born between 1994 and 1997 and then merged ZIP codes with < 100 children screened. To achieve the most homogeneous merged areas, we selected the adjacent ZIP code that had the closest expected percentage of elevated BLLs based on a regression model developed earlier (Talbot et al. 1998). This reduced the number of ZIP codes used in the analysis to 952.

The CDC defines an elevated BLL as ≥10 μg/dL (CDC 1997). We chose the percentage of children with elevated BLLs in each ZIP code group to be the dependent variable. There are many advantages of using this measure rather than the mean of the BLLs: Laboratories report results with different detection limits, extreme results may be due to child-specific traits such as pica, and we wanted to estimate the number of children with elevated BLLs in a community who will need follow-up blood lead screening and education. Log transformation of the dependent variable normalized the distribution.

We first used multiple linear regression to describe the relationship between children’s BLLs and the housing and socioeconomic variables. We analyzed the residual autocorrelation in the model using SpaceStat software (version 1.91; TerraSeer, Inc., Ann Arbor, MI). Spatial autocorrelation was expected because, according to Tobler’s first law of geography (Tobler 1970), “everything is related to everything else, but near things are more related than distant things.” Neighboring ZIP code groups may be similar because neighborhoods may cross ZIP code group boundaries and because children may visit or use services in nearby areas. Environmental and housing characteristics, secondary occupational exposures, and behavioral factors may also be similar in neighboring ZIP code areas. If two areas are very similar to each other, they provide less information to a model than do dissimilar areas. This causes the estimated standard deviations to be biased downward and results in reports of stronger levels of significance.

We then developed a simultaneous autoregressive model (SAR), as described by Anselin (1988). In this model, the predicted values are adjusted by the information in the surrounding ZIP codes. The SAR model can be written *Y* = *X*β+ ρ*W*(*Y* – *X*β) + ɛ. Here, *Y*, *X*, β, and ɛhave the same interpretation as in the linear regression model. The scalar ρ estimates the strength of the autocorrelation effect and can be interpreted similarly to the correlation coefficient: the closer its value is to 1, the stronger the positive autocorrelation between ZIP codes. W is the row-normalized weight matrix that describes the spatial structure of the data.

## Results

Table 1 summarizes statistics for the blood lead database by year. The final data set contained 677,112 children with blood lead tests, which represented 63.4% of children < 2 years of age born in 1994–1997. The screening rate was 65.6% if the lead records and births with missing ZIP codes were included. The percentage of children screened remained relatively constant during the study period, although it varied geographically. We compared the screening rates in poor inner-city neighborhoods with those of the rest of the state. The poor neighborhoods were defined as the upper fifth percentile of the variable “percentage of children living below the poverty level,” and they were located in certain ZIP codes in New York City, Buffalo, Rochester, Syracuse, Schenectady, and Albany. Eighty percent of the children were screened in poor inner-city neighborhoods, compared with 61% in other areas. The prevalence of children with elevated BLLs declined 44%, from 6.9% for children born in 1994 to 3.9% for children born in 1997. However, the rate of decline slowed in the last 2 years.

**Figure 1:**
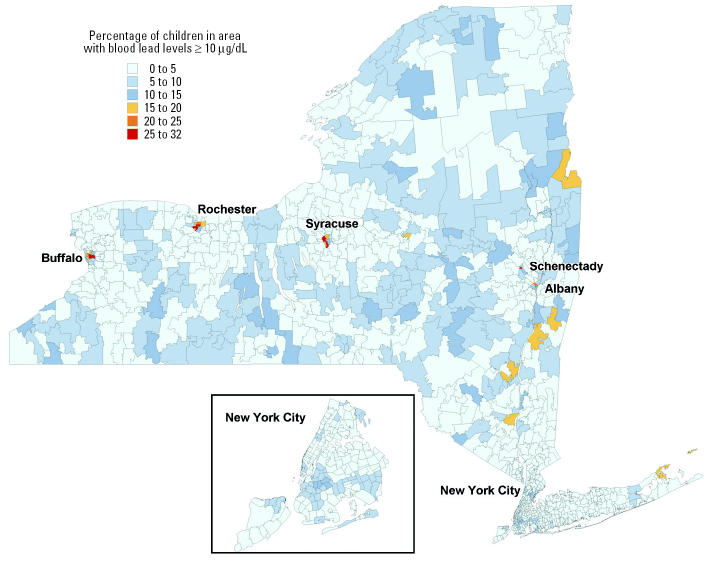
Prevalence of elevated BLLs of children born in 1994–1997 and screened before 2 years of age, by ZIP code groups containing at least 100 children screened.

The geographic distribution of the prevalence of elevated BLLs is shown in Figure 1. Most of the ZIP code areas had a small percentage of children with elevated BLLs. However, the upstate cities of Buffalo, Rochester, Syracuse, Albany, and Schenectady had some ZIP codes in which > 20% of the children screened had elevated BLLs. No ZIP code in New York City had > 15% of the children with elevated BLLs.

The linear regression model is summarized in Table 2. Because the effects of the independent variables in New York City were muted compared with the effects in the rest of the state, and none of the variables studied explained this difference, we developed a two-regime model. This model calculates separate parameters for New York City and for the rest of the state (upstate/Long Island) and presents one correlation coefficient for the whole model. Age of housing was the best predictor of the amount of children with elevated BLLs. The coefficient of determination (*r*^2^) for percentage of houses built before 1940 was 10% stronger than the coefficient for percentage of houses built before 1950, so we used the former. The poverty and education variables were highly correlated (*r* = 0.8). The effect of education was stronger in New York City, and the effect of poverty was stronger upstate. We chose to use only the education variable for consistency between models, because it was more linear than was the poverty variable and less correlated with the other variables in the model. The percentage of children born to African-American mothers remained significant after adjusting for age of housing and education. The other variables tested did not contribute strongly in both the New York City and upstate/Long Island regions. We excluded these variables because we wanted to create a parsimonious model and compare the same variables for each region.

The corresponding spatial error model is presented Table 2. When developing the spatial error model, four different weight matrices were tested: first-order neighbors, second-order neighbors, inverse distance to 25 km, and inverse distance squared to 25 km. First-order neighbors were chosen for the final model because they resulted in the best fit. There was a moderate amount of positive autocorrelation: ρ= 0.46. Modeling this correlation in adjacent ZIP codes widened the confidence intervals around the parameter estimates by 14% on average, and reduced the *r*^2^ by 21%. Spatial autocorrelation accounted for 11% of the variability in the data. The effects of housing age, education, and race were still stronger in the upstate/Long Island model than in the New York City model. The slopes of the parameter estimates were all within 20% of the multiple linear regression slopes.

**Figure 2:**
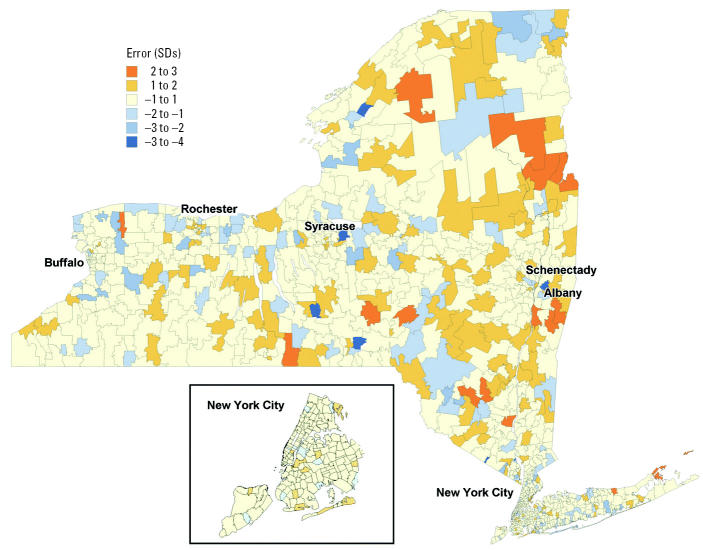
Residuals from SAR, by ZIP code groups containing at least 100 children screened.

The uncorrelated residuals for the SAR model are mapped in Figure 2. Areas where more children have elevated BLLs than the model predicts have positive residuals and are shown in orange. For example, there are some underpredicted ZIP code areas on the north fork of Long Island, the Hudson Valley, and eastern New York State, and there are some overpredicted ZIP codes (shown in blue) in the Bronx and the middle of Long Island, and western New York State.

**Figure 3:**
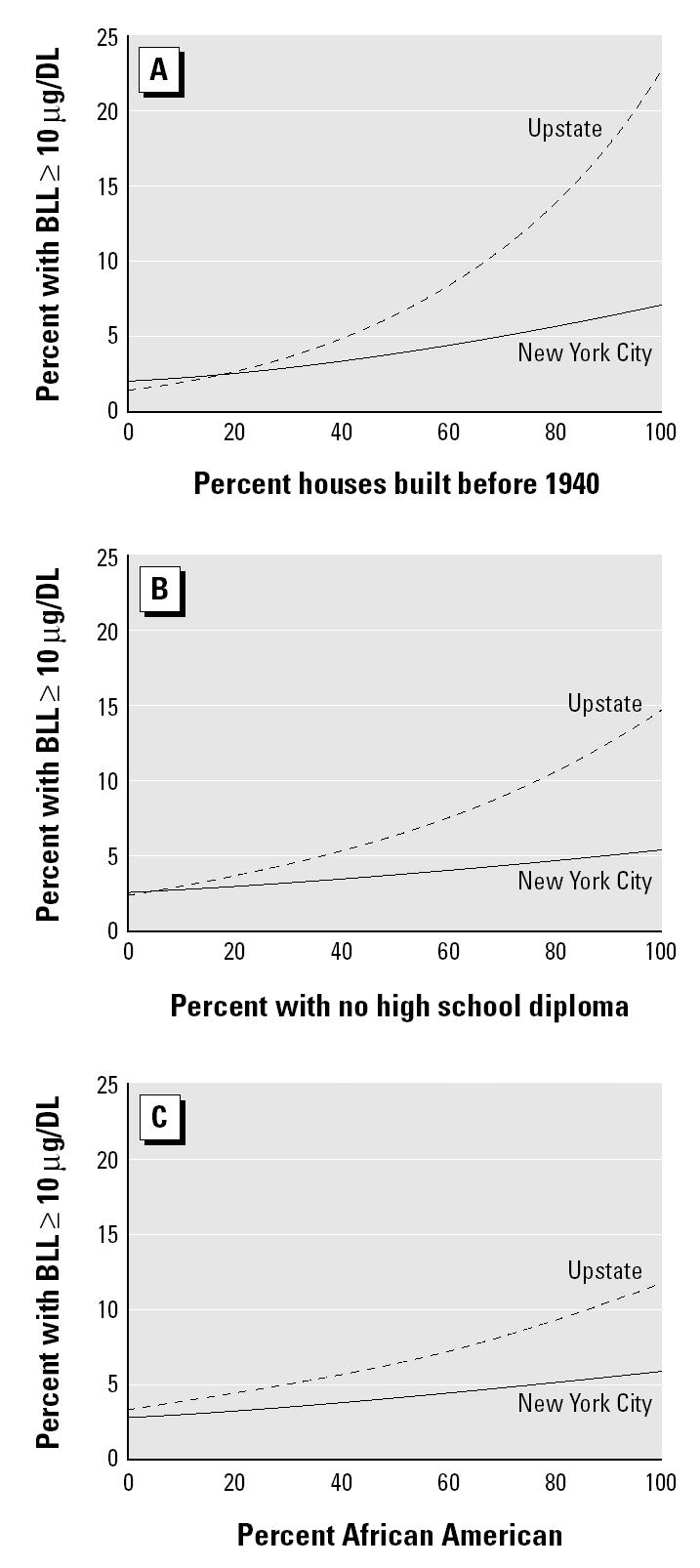
Conditional effect plots for the upstate/Long Island and New York City models. (*A*) Effect of age of housing. (*B*) Effect of education. (*C*) Effect of race. Mean value for percentage of homes built before 1940, 32%; percentage African American, 10%; percentage with no high school diploma, 23%.

Figure 3 contains conditional effect plots to facilitate interpretation of the slopes. These plots show the predicted value of elevated BLLs versus each of the variables used in the model while holding the other variables at their means and assuming an average autocorrelation of zero. They show that the effect of all the variables used in the model is weaker in New York City. We looked at the rates of rescreening to further examine reasons for the difference between New York City and the rest of the state. The CDC (1997) recommends that children with BLLs of 10–19 μg/dL be retested within 3 months and that those with higher BLLs be rescreened even sooner. Adherence to this recommendation was investigated by examining the first high test of all children up to 1.75 years of age so that their test results would be observed before 2 years of age; 27% of those in the 10–19 μg/dL range were retested within 3 months, and 62% of the ≥20 μg/dL range were retested within 3 months. The results were similar for New York City and the rest of New York State and thus do not contribute to the differences in these two areas of the state.

## Discussion

BLLs varied widely across the state. The age of housing, education level, and percentage of African-American births in a community were related to BLLs. These variables described a large portion of the variation in the data; the coefficient of determination we observed (*r*^2^ = 0.52) is very strong for an ecologic analysis.

The significance of housing, education, and race was consistent with other studies we reviewed. All researchers who tested it found age of housing to be significant. All researchers included a measure of socioeconomic status such as income or educational attainment in their final models. Educational attainment is often thought to be the most stable indicator of socioeconomic standing over the course of a lifetime, and it better captures persons not in the labor force such as homemakers. All the studies but one found that a higher proportion of African-American births was associated with elevated BLLs after controlling for housing and poverty. African Americans may have higher BLLs because of a lower calcium intake (Mahaffey et al. 1986) or poorer housing conditions (Lanphear et al. 1996). Sargent et al. (1997) found that the percentage of recent immigrants from other countries predicted elevated BLLs rather than percentage of African Americans, perhaps because the recent immigrants lived in worse neighborhoods, were less knowledgeable about lead hazards, or were exposed before immigration.

Some additional variables that other researchers included in their models were not appropriate for New York State because of large differences between New York City and the rest of the state. For example, we did not find housing tenure to be significant in New York City because renter-occupied housing is more common and may not relate to socioeconomic status in New York City as it does in the upstate/Long Island region. We found that the proportion of vacant homes, unadjusted by other census variables, did not capture the difference in how vacancy can exist in both impoverished neighborhoods and vacation areas. We found no association with population density. This is contrary to the findings of Lanphear et al. (1998), who reviewed data for Monroe County, New York, and those of Griffith et al. (1998), who looked at data for the city of Syracuse, New York. Within small geographic areas such as individual cities or counties, population density may be associated with other socioeconomic variables such as poverty. Across larger geographic areas, these associations become more complicated. We found that population density is not correlated with poverty in New York State because there are both rural and inner-city poor areas. The lack of association we found with population density is, however, consistent with earlier studies that looked at statewide data for New York (Talbot et al. 1998) and statewide data for Massachusetts (Sargent et al. 1995, 1997).

The percentage of children screened was positively associated with elevated BLLs in New York City and upstate cities but not in the rest of the state. We did not use screening rates in the model because the relationship between screening rate and BLLs often depends on the levels of environmental exposure and medical practices, which vary across the state. For example, in newer suburban areas where there is limited lead exposure, there would be a low percentage of children with elevated BLLs, regardless of the screening rate. In poor inner-city areas, where public assistance programs are successful at screening high-risk children, increasing the percentage screened to include those less at risk would decrease the percentage of children screened with elevated BLLs.

Although New York State regulation calls for universal screening of children at 1 and 2 years of age, we found that only 66% of New York State children born between 1994 and 1997 were screened before 2 years of age. However, this is the highest screening rate that we found in the literature. Surveys of pediatricians investigated reasons for non-compliance. In California, the physicians who did not test all patients tended to have less factual knowledge, less recent training, fewer minority patients, and less financial incentive (Ferguson and Lieu 1997). Nationwide, the noncompliant physicians did not commonly see children with elevated BLLs, and they believed that the benefits of screening did not outweigh the costs (Campbell et al. 1996). The U.S. General Accounting Office (GAO 1999) found that children who receive Medicaid are not being adequately screened. Although we did not relate our lead data to Medicaid data, we found that the screening rates were higher in poor inner-city neighborhoods, both in New York City and upstate. A further investigation of who is screened will require linking the lead records to birth certificates to obtain information on the race and educational attainment of the parents, and to real property records to obtain information on age and condition of housing.

The lower prevalence of elevated BLLs in New York City compared with upstate areas is surprising given that New York City is among the oldest and most densely populated areas of the state. New York City has a higher percentage of older housing stock and minority births and a lower percentage of high school graduates than does the rest of New York State. Based on these characteristics, some New York City neighborhoods would be expected to have some of the highest prevalence rates of elevated BLLs in the state. This was not the case, however, and none of the variables examined in this analysis could explain these findings. Several possibilities for the difference exist. New York City banned the residential use of lead paint in 1960, 18 years earlier than the rest of the state and nation (NYCDOH 1998), and began a lead poisoning prevention program earlier than did other areas (NYCDOH 2002). New York City also has a large number of public housing units. Regulations regarding the removal and abatement of lead-based paint in public housing have historically been stricter than for privately owned housing used by the poor (Chisholm et al. 1985). There may have been differences in abatement policies in New York State. Studies comparing blood lead data in two counties in New England found that children screened in an area with a historically weak abatement policy were more likely to have elevated BLLs than were children in an area that began actively enforcing an abatement policy 20 years before (Bailey et al. 1998; Sargent et al. 1999). Mielke (1999) speculates that the lower BLLs in Manhattan are due to the lack of exposed soil in this “concrete jungle.” This could explain Manhattan, but not the lower percentages in the entire region. Some residential areas in New York City are not very different in terms of green space from residential areas in upstate cities that have a much higher percentage of children with elevated BLLs. Water quality could also explain some of the difference. The New York City Water Department has been at the forefront of reducing lead in the water supply by reducing corrosivity (New York State Joint Legislative Commission 1992).

There are several possible limitations to this study. First, the children screened may not have represented the population. This could bias the parameter estimates. Additional bias could exist because of measurement error. For example, we analyzed the data at the ZIP code level, rather than using census tracks or census block groups that contain more homogeneous groups of people. We did not use census areas because it would be difficult and time-consuming to accurately assign the hundreds of thousands of children in our study to the correct census areas. Last, bias could be caused by missing variables, as follows.

Research suggests that soil lead exposure from both leaded gasoline and degraded lead paint is an important exposure pathway (Mielke and Reagan 1998). In Syracuse, New York, soil lead levels were associated with BLLs when areas were aggregated to the size of census tracts (Johnson and Bretsch 2002). Unfortunately, soil lead data are available only for a limited number of sites in New York State and thus cannot be incorporated into a statewide model. Mielke et al. (1997) explained that age of housing tends to overestimate the exposure of children in small towns because soil lead concentrations are low in old towns with low traffic flow. Historical land use can play an important role in the distribution of lead contamination in soil. For example, Eckel et al. (2001, 2002) located secondary lead smelters that were no longer operational and found high soil lead concentrations near the plants. These sites represent only a small fraction of industrial and commercial properties where lead was used. Housing condition is an important factor because children are more likely exposed to lead in old houses with deteriorating paint compared with well-maintained houses of the same age. Housing condition assessments may vary by town and are performed only from the outside of houses and on an irregular basis. There are also several unmeasured interventions that could have affected BLLs. For example, in select neighborhoods in eight New York State counties, the Healthy Neighborhoods Program went door to door to assess homes for the presence of lead paint hazards, provide educational materials, and ensure that children were appropriately screened. However, because this program covered such a small geographic area, it was not included in this analysis. The levels of funding for many housing programs that seek to reduce lead paint hazards also vary across the state. Because the types of incentives and reporting for these programs are so variable, we did not attempt to include them in the model. Air monitoring data were not included in the study because lead is monitored at only a small number of sites in New York State. The U.S. Environmental Protection Agency (EPA) National-Scale Air Toxics Assessment dispersion model was not used because it may not be reliable at the ZIP code level (U.S. EPA 2001).

## Conclusions

The dramatic 44% decline in elevated BLLs between children born in 1994 and 1997 is likely caused by greater public awareness, remediation and replacement of older homes, and the phaseout of lead from gasoline. The New York State data indicate that the rate of decline in the prevalence of elevated BLLs may be slowing. Similar declines have been observed nationwide. According to the National Health and Nutrition Examination Surveys, the percentage of children 1 to 5 years of age with elevated BLLs declined 50% between the 1991–1994 survey and the 1999–2000 survey (Meyer et al. 2003).

Lead remains an environmental health problem in inner-city neighborhoods, particularly in upstate New York. Older housing stock, a lower proportion of high school graduates, and a higher percentage of births to African-American mothers were the community characteristics most associated with elevated BLLs. Lead poisoning prevention resources should be targeted at the communities that we predicted to have a high prevalence or number of children with elevated BLLs.

We also identified areas with a higher or lower percentage of elevated BLLs than expected based on the model. These areas may have unique housing characteristics, other sources of environmental exposure, or differences in lead screening and remediation programs. These areas can be studied at the individual level to answer these remaining questions.

## Figures and Tables

**Table 1:** Summary children screened and those with elevated BLLs by year of birth.

	1994	1995	1996	1997	Total
No. (%) screened	169,395 (61.2)	172,986 (64.0)	171,890 (65.3)	162,841 (63.4)	677,112 (63.4)
No. (%) ≥10 μg/dL Pb	11,753 (6.9)	9,605 (5.6)	7,630 (4.4)	6,313 (3.9)	35,301 (5.2)

**Table 2:** Parameter estimates (SEs) for two-regime linear regression and spatial error models.

Variable*^a^*	Linear regression model	Spatial error model
New York City regime		
Intercept	0.8147 (0.0960)	0.8650 (0.1232)
Percent built < 1940	0.0110 (0.0017)	0.0102 (0.0018)
Percent without high school diploma	0.0059 (0.0024)	0.0060 (0.0030)
Percent African American	0.0069 (0.0012)	0.0060 (0.0014)
Upstate/Long Island regime		
Intercept	0.2926 (0.0414)	0.3675 (0.0521)
Percent built < 1940	0.0244 (0.0010)	0.0233 (0.0011)
Percent without high school diploma	0.0182 (0.0022)	0.0153 (0.0023)
Percent African American	0.0091 (0.0011)	0.0108 (0.0013)
ρ	NA	0.4649 (0.0383)
*r*^2^	0.63	0.52*^b^*

NA, not applicable.

a Dependent variable *Y* is ln(% elevated BLLs + 1) for both models.

b Pseudo *r*^2^ (Buse adjustment) used for spatial error model.

## References

[b1-ehp0112-001577] American Academy of Pediatrics (1998). Screening for elevated blood lead levels. Pediatrics.

[b2-ehp0112-001577] Anselin L 1988. Spatial Econometrics: Methods and Models. Dordrecht:Kluwer Academic Publishers.

[b3-ehp0112-001577] Bailey AJ, Sargent JD, Blake MK (1998). A tale of two counties: childhood lead poisoning, industrialization, and abatement in New England. Econ Geogr.

[b4-ehp0112-001577] Bailey AJ, Sargent JD, Goodman DC, Freeman J, Brown MJ (1994). Poisoned landscapes: the epidemiology of environmental lead exposure in Massachusetts children 1990–1991. Soc Sci Med.

[b5-ehp0112-001577] Brody DJ, Pirkle JL, Kramer RA, Flegal KM, Matte TD, Gunter EW (1994). Blood lead levels in the US population: phase 1 of the Third National Health and Nutrition Examination Survey (NHANES III, 1988 to 1991). JAMA.

[b6-ehp0112-001577] Campbell JR, Schaffer SJ, Szilagyi PG, O’Connor KG, Briss P, Weitzman M (1996). Blood lead screening practices among US pediatricians. Pediatrics.

[b7-ehp0112-001577] Canfield RL, Henderson CR, Cory-Slechta DA, Cox C, Jusko TA, Lanphear BP (2003). Intellectual impairment in children with blood lead concentrations below 10 μg per deciliter. N Engl J Med.

[b8-ehp0112-001577] CDC 1997. Screening Young Children for Lead Poisoning: Guidance for State and Local Public Health Officials. Atlanta, GA:Centers for Disease Control and Prevention.

[b9-ehp0112-001577] Chisholm JJ, Mellitis ED, Quaskey SA (1985). The relationship between the level of lead absorption in children and age, type, and condition of housing. Environ Res.

[b10-ehp0112-001577] Eckel WP, Rabinowitz MB, Foster GD (2001). Discovering unrecognized lead-smelting sites by historical methods. Am J Public Health.

[b11-ehp0112-001577] Eckel WP, Rabinowitz MB, Foster GD (2002). Investigation of unrecognized former secondary lead smelting sites: confirmation by historical sources and elemental ratios in soil. Environ Pollut.

[b12-ehp0112-001577] Ferguson SC, Lieu TA (1997). Blood lead testing by pediatricians: practice, attitudes, and demographics. Am J Public Health.

[b13-ehp0112-001577] Griffith DA, Doyle PG, Wheeler DC, Johnson DL (1998). A tale of two swaths: urban childhood blood-lead levels across Syracuse, New York. Ann Assoc Am Demogr.

[b14-ehp0112-001577] Johnson DL, Bretsch JK (2002). Soil lead and children’s blood lead levels in Syracuse, NY, USA. Environ Geochem Health.

[b15-ehp0112-001577] Landrigan PJ, Schechter CB, Lipton JM, Fahs MC, Schwartz J (2002). Environmental pollutants and disease in American children: estimates of morbidity, mortality, and costs for lead poisoning, asthma, cancer, and developmental disabilities. Environ Health Perspect.

[b16-ehp0112-001577] Lanphear BP, Byrd RS, Auinger P, Schaffer SJ (1998). Community characteristics associated with elevated blood lead levels in children. Pediatrics.

[b17-ehp0112-001577] Lanphear BP, Weitzman M, Eberly S (1996). Racial differences in urban children’s environmental exposures to lead. Am J Public Health.

[b18-ehp0112-001577] Litaker D Kippes CM Gallagher TE O’Conner ME 2000. Targeted lead screening: the Ohio lead risk score. Pediatrics 106(5):e69. Available: http://www.pediatrics.org/cgi/content/full/106/5/e69 [accessed 4 October 2004]. 10.1542/peds.106.5.e69 11061806

[b19-ehp0112-001577] Mahaffey KR, Gartside PS, Glueck CJ (1986). Blood lead levels and dietary calcium intake in 1- to 11-year-old children: the Second National Health and Nutrition Examination Survey, 1976 to 1980. Pediatrics.

[b20-ehp0112-001577] Meyer PA, Pivetz T, Dignam TA, Homa DM, Schoonover J, Brody D (2003). Surveillance for elevated blood lead levels among children—United States, 1997–2001. MMWR Surveill Summ.

[b21-ehp0112-001577] Mielke HW (1999). Lead in the inner cities. Am Sci.

[b22-ehp0112-001577] Mielke HW, Dugas D, Mielke PW, Smith KS, Smith SL, Gonzales CR (1997). Associations between soil lead and childhood blood lead in urban New Orleans and rural Lafourche Parish of Louisiana. Environ Health Perspect.

[b23-ehp0112-001577] Mielke HW, Reagan PL (1998). Soil is an important pathway of human lead exposure. Environ Health Perspect.

[b24-ehp0112-001577] Miranda ML, Dolinoy DC, Overstreet MA (2002). Mapping for prevention: GIS models for directing childhood lead poisoning prevention programs. Environ Health Perspect.

[b25-ehp0112-001577] Mushak P (1992). Defining lead as the premiere environmental health issue for children in America: criteria and their quantitative application. Environ Res.

[b26-ehp0112-001577] NYCDOH 1998. Childhood Lead Poisoning. City Health Information: Vol 17, no 2. New York:New York City Department of Health.

[b27-ehp0112-001577] NYCDOH 2002. Surveillance of Childhood Blood Lead Levels in New York City. New York:New York City Department of Health and Mental Hygiene.

[b28-ehp0112-001577] NYSDOH 2001. Protecting Our Children from Lead: The Success of New York’s Efforts to Prevent Childhood Lead Poisoning. Albany:New York State Department of Health.

[b29-ehp0112-001577] New York State Joint Legislative Commission on Toxic Substances and Hazardous Wastes 1992. Hearing Report: Lead Contamination in New York State, March 1992. Albany:New York State Joint Legislative Commission on Toxic Substances and Hazardous Wastes.

[b30-ehp0112-001577] Parsons PJ, Reilly AA, Esernio-Jenssen D (1997). Screening children exposed to lead: an assessment of the capillary blood lead fingerstick test. Clin Chem.

[b31-ehp0112-001577] Sargent JD, Bailey A, Simon P, Blake M, Dalton MA (1997). Census tract analysis of lead exposure in Rhode Island children. Environ Res.

[b32-ehp0112-001577] Sargent JD, Brown MJ, Freeman JL, Bailey A, Goodman D, Freeman DH (1995). Childhood lead poisoning in Massachusetts communities: its association with sociodemo-graphic and housing characteristics. Am J Public Health.

[b33-ehp0112-001577] Sargent JD, Dalton M, Demidenko E, Simon P, Klein R (1999). The association between state housing policy and lead poisoning in children. Am J Public Health.

[b34-ehp0112-001577] Talbot TO Forand SP Haley VB 1998. Geographic analysis of childhood lead exposure in New York State. In: Proceedings of the 3rd National Conference on GIS in Public Health (Williams RC, Hoiwe MM, Lee CV, Henriques WD, eds), 17–20 August 1998, San Diego, CA. Atlanta, GA:U.S. Agency for Toxic Substances and Disease Registry. Available: http://www.atsdr.cdc.gov/GIS/conference98/proceedings/proceedings.html [accessed 3 July 2003].

[b35-ehp0112-001577] Tobler W (1970). A computer movie simulating urban growth in the Detroit region. Econ Geogr.

[b36-ehp0112-001577] U.S. Census Bureau 1991. 1990 Census of Population and Housing: Summary File 1. Washington, DC:U.S. Department of Commerce.

[b37-ehp0112-001577] U.S. Census Bureau 1992. 1990 Census of Population and Housing: Summary File 3. Washington, DC:U.S. Department of Commerce.

[b38-ehp0112-001577] U.S. Census Bureau 2001. 2000 Census of Population and Housing: Summary File 1. Washington, DC:U.S. Department of Commerce.

[b39-ehp0112-001577] U.S. Census Bureau 2002. 2000 Census of Population and Housing: Summary File 3. Washington, DC:U.S. Department of Commerce.

[b40-ehp0112-001577] U.S. Consumer Product Safety Commission (1977). Notice reducing allowable levels of lead in lead-based paint. Fed Reg.

[b41-ehp0112-001577] U.S. EPA 2001. National-Scale Air Toxics Assessment for 1996, Preliminary Draft. EPA-453/R-01-003. Research Triangle Park, NC:U.S. Environmental Protection Agency.

[b42-ehp0112-001577] U.S. GAO 1999. Lead Poisoning; Federal Health Care Programs Are Not Effectively Reaching At-Risk Children. Report to the Ranking Minority Member, Committee on Government Reform, House of Representatives. GAO/HEHS-99-18. Washington, DC:U.S. General Accounting Office.

[b43-ehp0112-001577] Yiin L-M, Rhoads GG, Lioy PJ (2000). Seasonal influences on childhood lead exposure. Environ Health Perspect.

